# Vaccinia Virus Zoonotic Infection, São Paulo State, Brazil

**DOI:** 10.3201/eid1801.110692

**Published:** 2012-01

**Authors:** Jane Megid, Iara A. Borges, Jonatas S. Abrahão, Giliane S. Trindade, Camila M. Appolinário, Márcio G. Ribeiro, Susan D. Allendorf, João Marcelo A.P. Antunes, André T. Silva-Fernandes, Erna G. Kroon

**Affiliations:** Faculdade de Medicina Veterinária e Zootecnia–Universidade Estadual Paulista, Botucatu, São Paulo, Brazil (J. Megid, C.M. Appoliinário, M.G. Ribeiro, S.D. Allendoft, J.M.A.P. Antunes);; Universidade Federal de Minas Gerais, Belo Horizonte, Minas Gerais, Brazi (I.A. Borges, J.S. Abrahão, G.S. Trindade, A.T. Siilva-Fernandes, E.G. Kroon)

**Keywords:** vaccinia virus, viruses, zoonosis, Brazil

**To the Editor:** Since 1999, vaccinia virus (VACV) has been isolated frequently from dairy cattle and humans (*1**–**3*). During bovine vaccinia outbreaks, VACV can be transmitted to farmers and those who milk cows; it frequently causes lesions on the hands and forearms. Bovine vaccinia causes economic losses and affects public health services in Brazil (*1**–**4*). One of the first VACV viruses isolated during Brazilian bovine vaccinia outbreaks was Araçatuba virus (ARAV), which was collected in São Paulo State, and since that time, other VACVs have been isolated in this state (*2**,**5**,**6*).

The circulation of VACV in São Paulo forests was described in the 1960s and 1970s, although such isolates seem to be phylogenetically distinct from ARAV and other VACVs that currently circulate in Brazil (*7*). Although VACV in several Brazilian states has been reported (*1**–**3*), the intrastate spread of VACV concerns veterinary and medical authorities and presents a challenge to the sanitary barriers and prophylactic measures implemented to date. We report 2 zoonotic bovine vaccinia outbreaks in the midwestern region of São Paulo State, Brazil.

The Institutional Ethics and Animal Welfare Commission from the Faculdade de Midicina Veterinária e Zootecnia–Universidade Estadual Paulista Júlio de Mesquita Filho/Campus de Botucatu approved this study. In 2009 and 2010, exanthematic outbreaks were reported in rural areas of Itatinga (23°6′7′′S, 48°36′57′′W) and Torre de Pedra (23°14′38′′S, 48°11′42′′W) counties, respectively. Between the 2 outbreaks, lesions were observed on the teats and udders of 10 lactating cows. The lesions appeared as macules, evolved into vesicles, pustules, and ulcers and healed after 2–3 weeks. Lesions developed on the hands and arms of the milkers after occupational contact with sick animals. The milkers also described headache, lymphadenopathy, and fever.

Specimens from 7 scabs and 1 vesicle were collected for virus identification by laboratory assays. After DNA extraction (InvitekDNA, Berlin, Germany), the samples were subjected to a specific orthopoxvirus PCR for the amplification of the A56R gene of vaccinia virus (*8*). A fragment of ≈950 bp was amplified from 5 exanthematic lesions. Two milk samples collected from sick cows were also positive for A56R. Parapoxvirus DNA was not detected in any collected sample (*9*). Material from the bovine and human exanthematic lesions induced characteristic poxvirus cytopathic effects in baby hamster kidney cells. In addition, 13 of the 18 collected bovine serum specimens were positive for orthopoxvirus according to a plaque reduction neutralization test and an ELISA (*4*). Human serum specimens were negative for orthopoxvirus by the plaque reduction neutralization test but positive by IgM ELISA, indicating the occurrence of an acute infection process (*4*).

A56R-PCR amplicons from 2 exanthematic lesions and 2 milk samples were sequenced in both orientations by using the Mega-BACE-sequencer (GE Healthcare, Little Chalfont, UK). Optimal alignment of our samples and other orthopoxvirus A56R gene sequences with ClustalW (www.ncbi.nlm.nih.gov/pmc/articles/PMC308517) by using MEGA3.1 (www.megasoftware.net) showed that a signature deletion was present in the sequences of several Brazilian VACV isolates (*1*–*3*). Three of the 4 sequenced amplicons exhibited 100% identity: the milk samples and a lesion collected from a same county. VACV samples from Itatinga and Torre de Pedra showed high identity with ARAV (*2*) and other Brazilian VACVs, including the Cantagalo (*1*) and Mariana viruses (*10*). A phylogenetic tree based on the A56R gene was constructed with the neighbor-joining method, 1,000 bootstrap replicates, and the Tamura 3-parameter model (MEGA3.1) (Figure). VACVs from Itatinga and Torre de Pedra clustered with several VACVs isolated during bovine vaccinia outbreaks. The A56R sequences obtained in this study were deposited in GenBank (accession no. It1446645).

**Figure:**
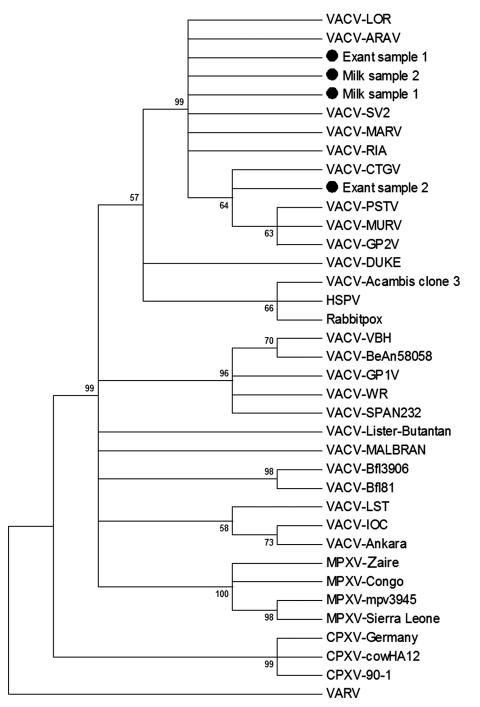
Consensus bootstrap phylogenetic tree based on the nucleotide sequences of the A56R-hemagglutinin gene of vaccinia virus. The tree was constructed with hemagglutinin sequences by using the neighbor-joining method with 1,000 bootstrap replicates and the Tamura 3-parameter model in MEGA3.1 software (www.megasoftware.net). Bootstrap values >50% are shown. Nucleotide sequences were obtained from GenBank. Black dots indicate the vaccinia virus (VACV) analyzed in this study. HSPV, horsepoxvirus; VARV, variola virus; CPXV, cowpoxvirus; MPXV, monkeypoxvirus.

We describe a new zoonotic outbreak of bovine vaccinia in São Paulo State, Brazil. Our molecular data suggest that this outbreak was caused by a VACV that is genetically related to viruses isolated in previous years, including ARAV, which was isolated in 1999 (*2*). The emergence and reemergence of this virus in previously bovine vaccinia–free microregions of São Paulo State suggest that VACV could have adapted to a specific microbiome and that the virus may be circulating not only in cattle and humans but also in some wild reservoir (*10*). Although genetic and ecologic studies of Brazilian VACVs have advanced in the past several years, little has been achieved in terms of bovine vaccinia prevention and control. Therefore, bovine vaccinia surveillance and public communication are critical in areas where VACV circulates.
